# Primary Signet Ring Cell Adenocarcinoma of the Appendix Masquerading as Acute Appendicitis: A Diagnostic Pitfall

**DOI:** 10.22034/ijp.2026.2069876.3520

**Published:** 2026-08-01

**Authors:** Ankita Singh, Kanwarpreet Kaur Cheema, Shikhar Chohan, Hanisha Jain, Praphulla Chandra Prabhakar

**Affiliations:** 1 Department of Pathology, Sanjay Gandhi Memorial Hospital, New Delhi, India; 2 Department of Pathology, Vardhman Mahavir Medical College & Safdarjung Hospital, New Delhi, India

**Keywords:** Appendix, Adenocarcinoma, Signet Ring Cells

## Abstract

**Background & Objective:**

Primary signet ring cell adenocarcinoma (PSRCA) of the appendix is a rare and aggressive malignancy accounting for approximately 4% of all appendiceal neoplasms. These tumors often present at an advanced stage with nonspecific clinical and radiological findings and frequently mimic acute appendicitis. Hence, histopathological evaluation remains essential for definitive diagnosis.

**Case Presentation:**

A 64-year-old female presented with clinical features suggestive of acute appendicitis. Radiological evaluation revealed appendicolithiasis. The patient underwent appendectomy, and histopathological examination demonstrated a signet ring cell adenocarcinoma diffusely infiltrating through the appendiceal wall into the mesoappendix (pT3). Lymphovascular invasion was present. Both the proximal and mesenteric margins were positive for tumor infiltration. No lymph nodes were submitted in the specimen. Immunohistochemistry showed tumor cells positive for CDX2, CK20, and MUC-1 and negative for CK7, supporting a primary lower gastrointestinal tract origin and favoring an appendiceal origin.

**Conclusion:**

PSRCA of the appendix is an aggressive malignancy often presenting at an advanced stage and mimicking acute appendicitis. Accurate histopathological evaluation is essential, as the diagnosis has important therapeutic implications, including the need for further oncological management beyond appendectomy.

## Introduction

Primary signet ring cell adenocarcinoma (PSRCA) of the appendix is an exceedingly rare and highly aggressive malignancy(1,2). It is characterized by diffuse infiltration, early transmural spread, and a poor prognosis(2,3). These tumors are common in the elderly age group and, clinically and radiologically, often present with nonspecific features and commonly mimic acute appendicitis, leading to an initial misdiagnosis(2,4).

Due to its rarity and overlapping presentation with benign inflammatory conditions, PSRCA is frequently diagnosed incidentally on histopathological examination of appendectomy specimens(1,2). Accurate diagnosis is crucial, given its significant impact on treatment and prognosis, and it often requires additional surgical and therapeutic intervention(3,5).

This report aims to highlight a rare case of primary signet ring cell adenocarcinoma of the appendix presenting as acute appendicitis and emphasize its diagnostic and clinical significance.

## Case Presentation

A 64-year-old female presented to the surgical OPD with complaints of right lower abdominal pain for 7 days. Baseline hematological and biochemical investigations were within normal limits. CT scan revealed a prominent appendix with multiple small appendicoliths, suggestive of appendicolithiasis.

**Fig. 1:**
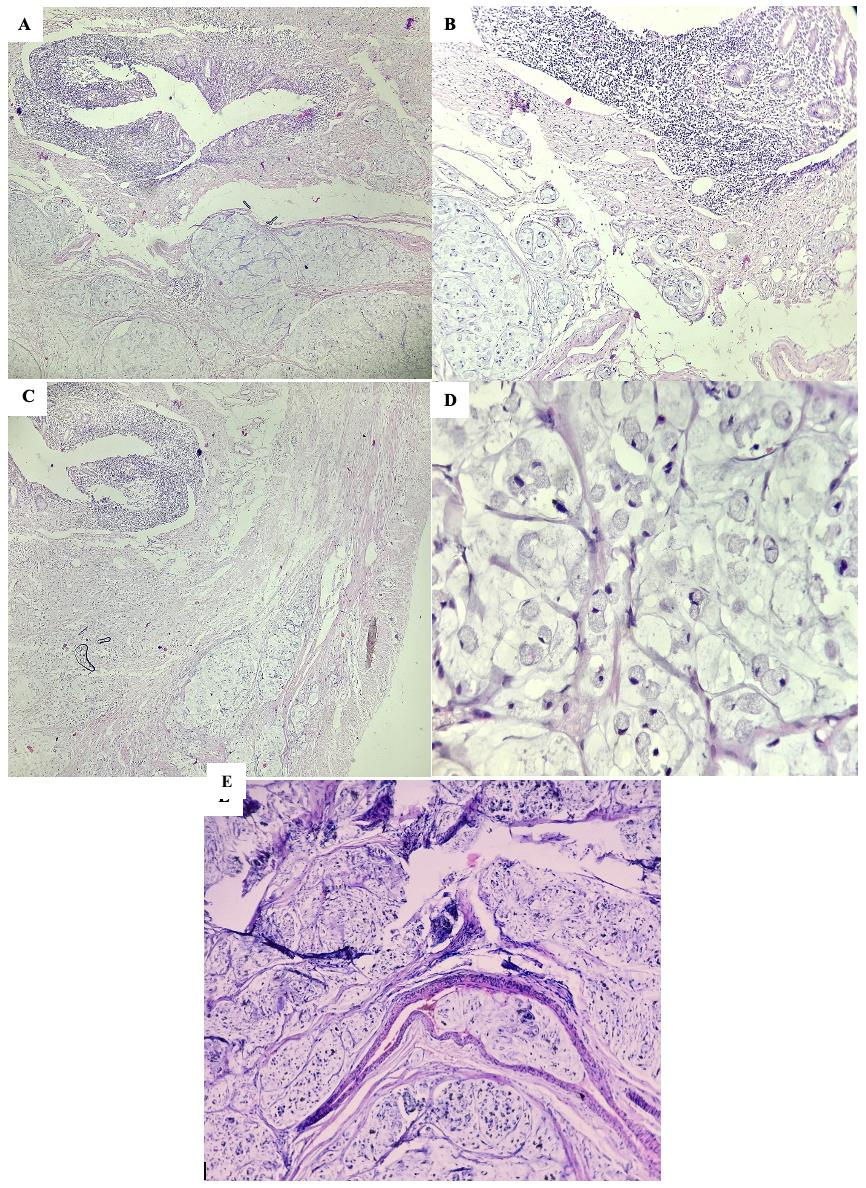
A: A scanner view showing islands of signet ring cells invading the appendix circumferentially (H&E, x10); B: Low power view of the same (H&E, x10); C: Section showing invasion of muscularis propria (H&E, x10); D: High power view of tumor cells (H&E, x40); E: Lymphovascular invasion (H&E, x40)

The patient underwent appendectomy, and the specimen was submitted for histopathological examination (HPE). Grossly, the appendix measured 1.8 cm in length and 0.6 cm in diameter. The serosal surface appeared congested and showed a perforation measuring 0.6 cm in greatest dimension. On cut section, the appendiceal wall was diffusely thickened, firm, and grayish white. The lumen was dilated and filled with mucinous material. The tumor size could not be precisely assessed due to extensive involvement. The tumor was grossly approaching both surgically resected margins (Fig. 1).

## Discussion

Primary signet ring cell carcinoma (SRCC) of the appendix is an exceedingly rare and aggressive malignancy, often presenting with nonspecific clinical features mimicking acute appendicitis, thereby posing significant diagnostic challenges(6). Owing to its rarity and overlapping histomorphology, accurate diagnosis requires integration of histopathological findings, immunohistochemistry, and clinicoradiological correlation(1,2).

**Fig. 2:**
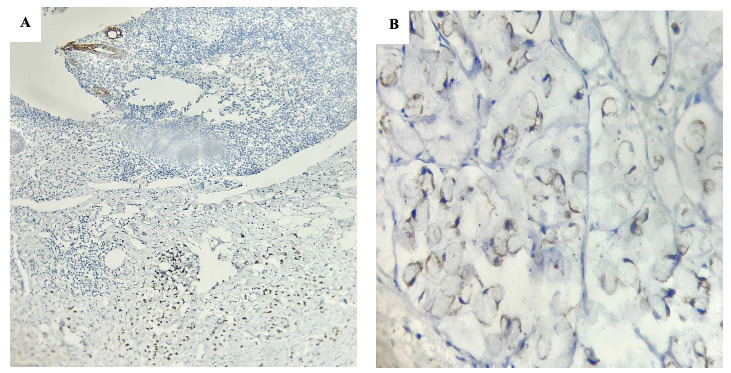
Immunohistochemical examination showing immunoreactive epithelial cell and signet ring cells for CK20. A: The epithelial cells show diffuse strong immunoreactivity for CK 20. B: Signet ring cells show strong membranous positivity for CK20.

**Fig. 3:**
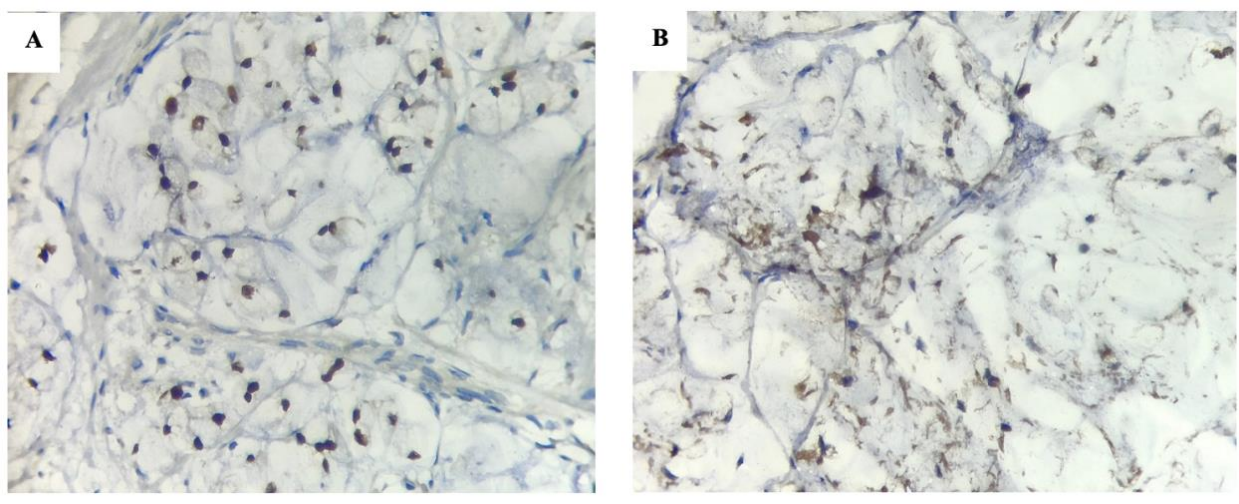
A Signet ring cells show strong nuclear stain for CDX2. B Tumor cells are immunoreactive for MUC-1.

**Fig. 4:**
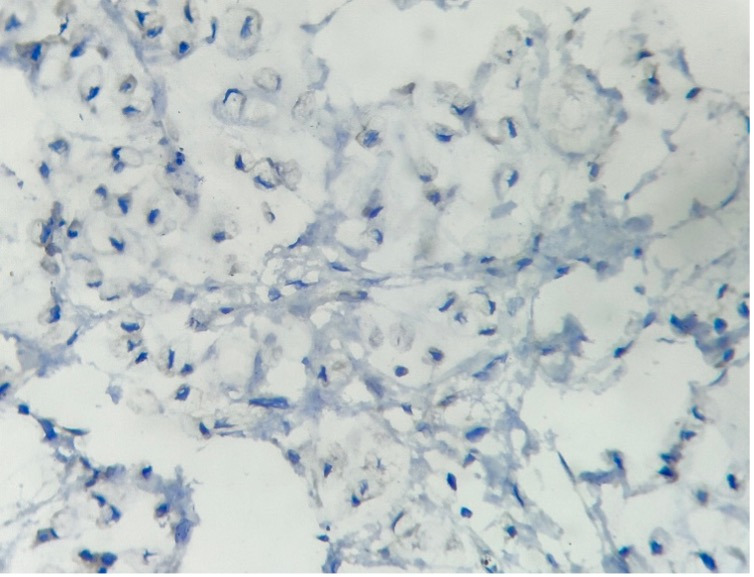
Tumor cells are negative for CK7.

A key diagnostic consideration is the exclusion of metastatic SRCC, particularly from the stomach, followed by colorectal primaries. This distinction is critical, as it directly influences staging, management, and prognosis. Immunohistochemical markers such as CDX2 indicate intestinal differentiation but lack site specificity and should not be used in isolation. A panel-based approach including CK7, CK20, and mucin markers (MUC-1, MUC-2, MUC-5AC) improves diagnostic accuracy when interpreted in conjunction with clinical and imaging findings(5,6,7). In the present study, tumor cells were positive for CDX2, CK20, and MUC-1 and negative for CK7 (Figs. 2–4).

Frozen-section evaluation may be limited by sampling constraints and subtle cytological features of signet ring cells, thereby increasing the risk of underdiagnosis. Therefore, definitive diagnosis should rely on a comprehensive evaluation of adequately sampled formalin-fixed tissue(8).

From a clinical perspective, colonoscopy and cross-sectional imaging are essential to exclude a primary tumor elsewhere and to assess disease extent(7,9). Given the aggressive nature and poor prognosis, accurate staging is crucial.

Various recent studies indicate that appendiceal SRCC is frequently diagnosed incidentally following appendectomy and is often associated with advanced disease at presentation, including transmural invasion and peritoneal dissemination(6,7). Prognostic studies have demonstrated that adverse outcomes are strongly associated with higher T stage, serosal involvement, and positive resection margins, reflecting the infiltrative nature of this tumor subtype(10). Imaging is often nonspecific, further contributing to delayed or incidental diagnosis(9).

In the present case, the tumor size could not be precisely assessed due to extensive involvement. The tumor cells demonstrated invasion through the muscularis propria and involvement of the mesoappendix, corresponding to an advanced local stage (pT3). Additionally, resection margins involved by tumor represent a significant adverse prognostic factor and raise concern about residual disease. These findings are consistent with recent literature describing the aggressive behavior and poor prognostic profile of SRCC, even in relatively small tumors due to their diffuse infiltrative growth pattern(10).

Although lymph node status was not available, the presence of mesoappendix involvement and margin positivity places the patient in a high-risk category, warranting further clinical evaluation and consideration of additional surgical and oncological management. However, the lack of data in the present case precludes assessment of treatment response and survival outcome.

These findings underscore the importance of complete pathological staging, margin assessment, and longitudinal follow-up in such rare malignancies to enable accurate prognostication and meaningful comparison with emerging literature.

## Conclusion

PSRCA of the appendix represents an exceedingly rare and aggressive malignancy, often associated with poor prognosis due to its insidious presentation and advanced stage at diagnosis. Preoperative laboratory investigations and imaging studies are usually noncontributory. Thus, meticulous HPE is essential for establishing the diagnosis. IHC further serves as a valuable adjunct in confirming the appendiceal origin.

Given the rarity of such cases, comprehensive documentation is essential, not only to broaden the clinicopathological spectrum but also to emphasize the need for newer management approaches aimed at improving clinical outcomes and overall prognosis.

## Abbreviations

PSRCA: Primary Signet Ring Cell Adenocarcinoma

OPD: Outpatient Department

CT: Computed Tomography

HPE: Histopathological Examination

H&E: Hematoxylin and Eosin

IHC: Immunohistochemistry

CK20: Cytokeratin 20

MUC-1: Mucin 1

CK7: Cytokeratin 7

SRCC: Signet ring cell carcinoma.

## Data Availability

The data that support the findings of this study are available from the corresponding author upon reasonable request.
